# “He is suitable for her, of course he is our relative”: a qualitative exploration of the drivers and implications of child marriage in Gezira State, Sudan

**DOI:** 10.1136/bmjgh-2018-001264

**Published:** 2019-05-28

**Authors:** Laura Dean, Angela Obasi, Asma El Sony, Selma Fadul, Hanaa El Hassan, Rachael Thomson, Rachel Tolhurst

**Affiliations:** 1 Department of International Public Health, Liverpool School of Tropical Medicine, Liverpool, UK; 2 Epidemiological Laboratory, Epi-Lab, Khartoum, Sudan; 3 Department of Tropical Disease Biology, Liverpool School of Tropical Medicine, Liverpool, UK

**Keywords:** qualitative study, child health

## Abstract

**Introduction:**

Child marriage is a fundamental development challenge for women and girls, with significant negative health and social outcomes. Sudan has a high rate of child marriage, with 34% of women aged 20–24 married before their 18th birthday. Since limited preventive interventions exist, we aimed to inform the evidence base to strengthen strategic action, using mixed qualitative methods to enhance study credibility. This study is the first to conduct a rigorous qualitative examination of the drivers of child marriage from the perspective of key stakeholders involved in marriage decision making within Sudan, and makes a significant contribution towards global knowledge by developing an evidence-based conceptual framework.

**Methods:**

Initially, we completed 14 focus group discussions separated by gender with mothers, fathers, and girls married as adolescents, and 23 key informant interviews. We then used a critical incident case study approach to explore 11 ‘cases’ of child marriage (46 interviews).

**Results:**

Findings indicate that gendered social norms and values, underpinned by religious beliefs and educational accessibility, interconnect to shape marriage decisions. In this *context*, many child marriages are *triggered* by an intrakinship proposal and further enabled by the relative lack of autonomy and influence of girls and women in marriage decision-making *processes*.

**Discussion:**

Interconnected drivers demand context-specific holistic and multisectoral approaches, which should include simultaneous strategies to expand access to education, health services and livelihood opportunities, and evoke legal change, and participatory social and attitudinal processes that include the engagement of religious leaders and men.

**Key questions**

**What is already known?**

- Child marriage is a fundamental development challenge for women and girls, with significant negative health and social outcomes.
- The evidence base is growing on the causes and consequences as well as the efficacy of various intervention models.
- Minimal research has explored holistically how social and structural drivers converge to perpetuate ongoing cycles of child marriage particularly in the Eastern Mediterranean region.

**What are the new findings?**

- Marriage decisions in Gezira State, Sudan, are shaped by patriarchal gendered norms, religion and challenges to educational access, which when triggered by an intrakinship proposal can result in child marriage, as well as a lack of of autonomy for women and girls in marriage decision making, leading to a process of ‘manufactured consent’.

**What do the new findings imply?**

- Interconnected drivers demand holistic and multisectoral intervention approaches to increase marriage age and to improve the health and well-being of married girls.

## Introduction

Child marriage, defined as any ‘legal or customary union involving a boy or girl below the age of 18’,^1^ disproportionately affects girls, with 12 million girls under 18 currently marrying each year.^2^ Child marriage is a violation of human rights and presents significant health challenges for girls and women. It is a major determinant of adolescent pregnancy, the single largest cause of mortality among girls aged 15–19 in low-income and middle-income countries.^3^ Child marriage limits educational and economic prospects, has been linked with increased physical, sexual and emotional violence, and can lead to social isolation and disempowerment.^4–6^ Such impacts of child marriage have been found to be both direct and intergenerational at the individual and societal levels.^7^

Islamic countries have some of the highest rates of child marriage in the world.^8^ Within the poorest countries in the Eastern Mediterranean region, such as Somalia, over 40% of girls marry before their 18th birthday.^9^ Although rates of child marriage in the region have generally declined over the last generation, this decline has recently stagnated and has stopped completely in some countries.^9^ Sudan has one of the highest prevalence of child marriage within the region, with 34% of women aged 20–24 married before their 18th birthday, compared with an 18% regional average.^2 10^

Increasing international recognition of child marriage as a fundamental development challenge has led to growing evidence on its causes and consequences,^1^ and the efficacy of various intervention models for preventing or reducing the practice.^5^ However, the current evidence base is disparate, lacking in contextual diversity and heavily reliant on ‘grey literature’. Limited high-quality evaluations of intervention effectiveness to date suggest the need to further interrogate the drivers of child marriage to improve intervention design in specific settings. Minimal research has explored holistically how social and structural drivers converge to perpetuate ongoing cycles of child marriage in the Eastern Mediterranean region.

This study aimed to understand drivers of child marriage within Gezira State, Sudan. Specific objectives were (1) to explore sociocultural and economic norms and drivers relating to marriage practices among adolescent girls (10–19) from the perspective of adolescent girls, their families and key community stakeholders; (2) to understand the pathways, decision-making processes and triggers of marriage among adolescent girls from the perspective of adolescent girls, their parents and other influential individuals; and (3) to infer how drivers of child marriage in this context may influence the applicability of interventions to address child marriage and improve women’s and girls’ health and well-being.

## Methods

### Study setting

Sudan is an Islamic state where approximately 97% of the population are Muslim. The state of Gezira, in east-central Sudan, has a highly dispersed population of approximately three million.^10^ The state of Gezira was selected for this study due to its accessibility both geographically and based on existing partner relationships. Given the sensitive nature of the research topic, having an established relationship with the Gezira Ministry of Health as well as existing relationships with Non-Governmental Development Organisations (NGDOs) operating in the area was essential to facilitate community access and acceptance, as well as offering opportunities to develop and implement interventions. Gezira is one of Sudan’s most developed states with a literacy rate of 59.2% among women aged 15–24, compared with a national average of 45.2%.^10^ Most Geziran households (44.5%) fall within the second highest wealth quintile.^10^ The prevalence of child marriage (women aged 15–19 years who are currently married) is 21% in the state of Gezira. Comparatively, the state of Khartoum has the lowest prevalence of child marriage at 12% and the state of Gadarif has the highest at 33.1%. The majority of other states have a prevalence of between 20% and 30%.^11^

### Study design, sampling and data collection

**Figure 1:**
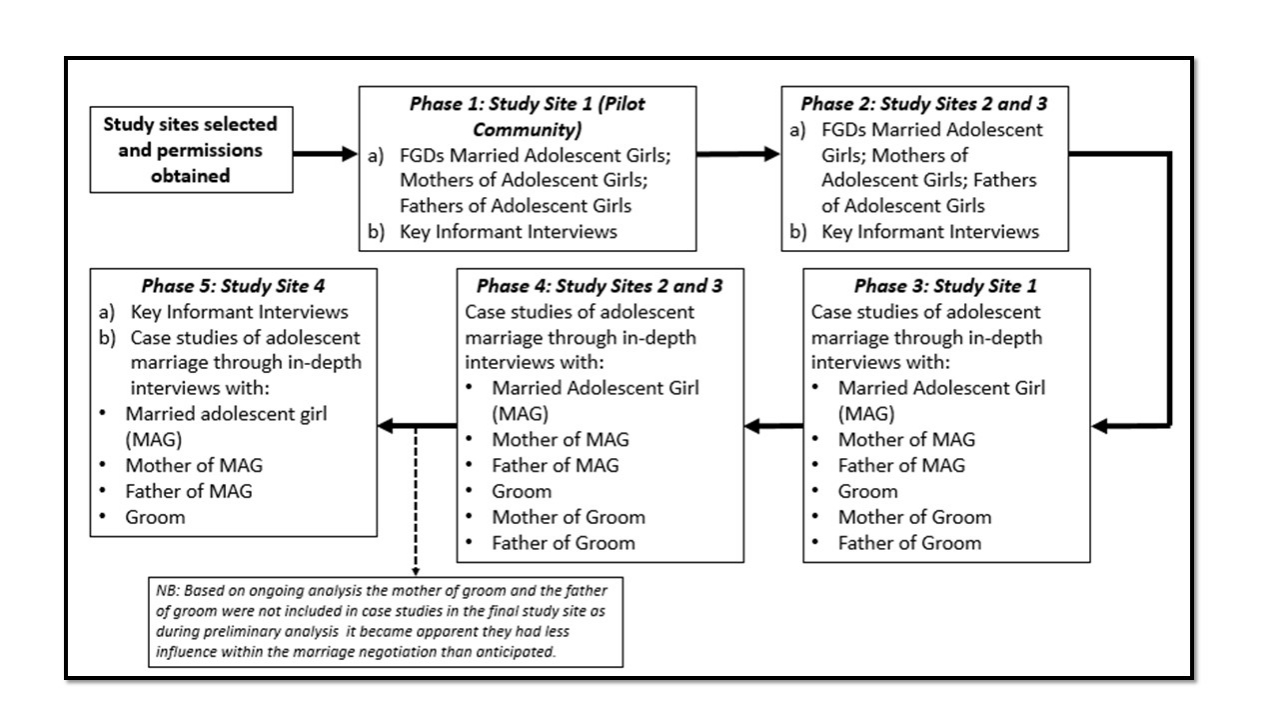
Phased study design. FGD, focus group discussion.

We conducted phased qualitative research between October 2014 and March 2016 in four villages in rural Gezira (figure 1). Study localities were purposively selected to represent average population size and levels of health, education and water service provision. Variation in ethnicity was aimed for across the sample. Study villages were further purposively selected from a sampling frame of all villages in selected localities, aiming for maximum variation^12^ in village characteristics (table 1). Villages with a high secondary school were excluded, as this was rare across localities.

**Table 1:** Study site characteristics

	Population	Primary school	Nearest high secondary school (vehicle, dry season) (min)	Formally trained midwife	Healthcare worker	Distance to Primary Health Care (PHC) (vehicle, dry season) (min)	Distance comprehensive Emergency Obstetric Care (EmOC) (vehicle, dry season)	Key ethnic groups
Study site 1	4300	Yes	10	Yes	Medical assistant	NA	20 min	Khawalda
Study site 2	3750	Yes	15	No	No	15	45 min	Kawahla and Ga’leeyen
Study site 3	3250	Yes	30	No (traditional midwife)	Retired nurse	30	1 hour	Kawahla and Ga’leeyen
Study site 4	1415	Yes	10	Yes	Nurse	45	45 min	Kawahla, Ga’leeyen, Shukreya and Bataheen

NA, not available.

We combined three methods to explore perspectives among men, women and adolescent girls (figure 1). Initially, 14 focus group discussions (FGDs) (3 villages) and 23 key informant interviews (KII) (4 villages) explored perceptions and social norms around aspirations for girls, marriage of adolescent girls, perceived benefits and risks of girls’ education, and potential strategies for improving their health and well-being. FGDs were conducted separately with girls married as an adolescent and currently under the age of 25, mothers of adolescent girls (aged 10–19) and fathers of adolescent girls. KIIs were conducted with influential community members in all four villages, including the Imam, Imam’s wife, midwives/health personnel, teachers and community elders. FGDs and KIIs concluded once thematic saturation was reached.

We explored 11 ‘cases’ of child marriage (46 interviews) using a critical incident case study approach, comprising indepth interviews (IDIs) with up to six individuals identified during FGD and KII as ‘key actors’ within the marriage process: girl married as an adolescent (MAG) (always included), husband, father of MAG, mother of MAG, father of the husband and mother of the husband. Interviewees were asked to reflect on the ideals of marriage and education for girls, actual pathways for girls’ education, decision making about the case study marriage, and health and well-being outcomes.

To identify child marriage ‘cases’, a sample frame of all girls married before or at 18 and currently under the age of 25 was generated in collaboration with key informants in each community. ‘Cases’ were purposively selected to achieve maximum variation in age at marriage as this may cause variance in drivers and outcomes.^13–15^ In all cases, husbands were between 7 and 11 years older than MAGs (table 2).

**Table 2:** Marriage case study participant details

	Type of respondent*†	Approximate age at interview‡	Approximate age at marriage‡	Occupation	Educational level
	Study site 1 (SS1)				
Case study 1 (CS1)	MAG	18	15	Housewife	Primary
	Groom	25	22	Builder	Primary
	MoB	Unknown		Housewife	Primary
	FoB	35		Builder	Primary
	MoG	Unknown		Farmer	Primary
	FoG	54		Farmer	Primary
	Study site 2 (SS2)				
Case study 2 (CS2)	MAG	20	18	University student	University
	Groom	28	26	Farmer and trader	University
	MoB	50		Housewife	No education
	FoB	58		Driver	No education
	MoG	45		Housewife	No education
	FoG	56		Farmer and trader	Primary
Case study 3 (CS3)	MAG	21	18	Housewife	Secondary
	Groom	27	24	Trader	Secondary
	MoB	45		Housewife	No education
	FoB	50		Bed maker	Primary
	MoG	45		Housewife	No education
	FoG	59		Trader	Primary
Case study 4 (CS4)	MAG	20	18	Housewife	University
	Study site 3 (SS3)				
Case study 5 (CS5)	MAG	18	18	Housewife	Secondary
	Groom	27	27	Animal farmer	Primary
	MoB	43		Housewife	No education
	FoB	53		Farmer	No education
	MoG	48		Housewife	No education
Case study 6 (CS6)	MAG	18	17	Housewife	Primary school
	Groom	27	26	Farmer	Primary school
	MoB	40		Housewife	Primary school
	FoB	46		Shepherd	Primary school
	MoG	55		Housewife	No education
	FoG	70		Farmer	No education
	Study site 4 (SS4)				
Case study 7 (CS7)	MAG	18	17	Housewife	Primary
	Groom	26	25	Layman	Primary
	MoB	45		Housewife	No education
	FoB	45		Builder	Primary
Case study 8 (CS8)	MAG	16	15	Housewife	Primary
	Groom	25	24	Merchant	Secondary
	MoB	45		Housewife	No education
	FoB	50		Village health assistant	Primary
Case study 9 (CS9)	MAG	15	14	Housewife	Primary
	Groom	26	25	Shopkeeper	Secondary
	MoB	45		Housewife	No education
Case study 10 (CS10)	MAG	15	14	Housewife	Primary
	Groom	23	22	Shopkeeper	Secondary
	MoB	25		Housewife	Primary
Case study 11 (CS11)	MAG	18	16	Housewife	Primary
	Groom	25	23	Trader	Secondary

*Type of respondent acronyms as follows: married adolescent girl (MAG), groom, mother of bride (MoB), father of bride (FoB), mother of groom (MoG) and father of groom (FoG).

†Where expected participants are missing from case studies, participants were either deceased, not living in study communities or refused to take part in the study.

‡Ages represented may not be accurate as participants were often unaware of their age and so gave an approximation. This was common across all participants.

Topic guides were developed for all FGDs, KIIs and IDIs in English, translated into Arabic, back-translated and further modified after piloting in the first study community. Written informed consent was obtained from all participants prior to data collection. Where participants were illiterate, information sheets and consent forms were read out to them and a thumb print taken to represent a signature. All FGDs, KIIs and IDIs lasted between 60 and 90 min, were audio-recorded, and conducted in Arabic by SF and a team of other locally based trained researchers, including one male and six female researchers. Data were transcribed in Arabic, translated into English and a sample back-translated to check for quality.

### Study participant characteristics

Most MAGs described themselves as a housewife, while husbands reported various formal and informal livelihood activities. Few participants were educated above primary school level. However, four husbands had achieved secondary education, and two wives and one husband had achieved university-level education. A wife was more educated than her husband in one case study (case study 5).

### Data analysis

Data were analysed thematically using a framework approach, detailed as follows.^16^ Research team members familiarised themselves with the data and developed an inductive coding framework, iteratively refined throughout analysis. Data were coded by LD and quality-checked by RTo. Interviews were analysed thematically across the full data set as well as grouped by marriage case study to allow for exploration of similarities and difference in key actors’ accounts of the marriage. Data were charted based on emerging themes across and between codes. LD, RTo and SF wrote a descriptive account of the data based on these charts. All authors reviewed and discussed the descriptive account to explore the range, diversity and links between and within themes and used this as a basis for generating an explanatory account.

### Community dissemination

**Figure 2:**
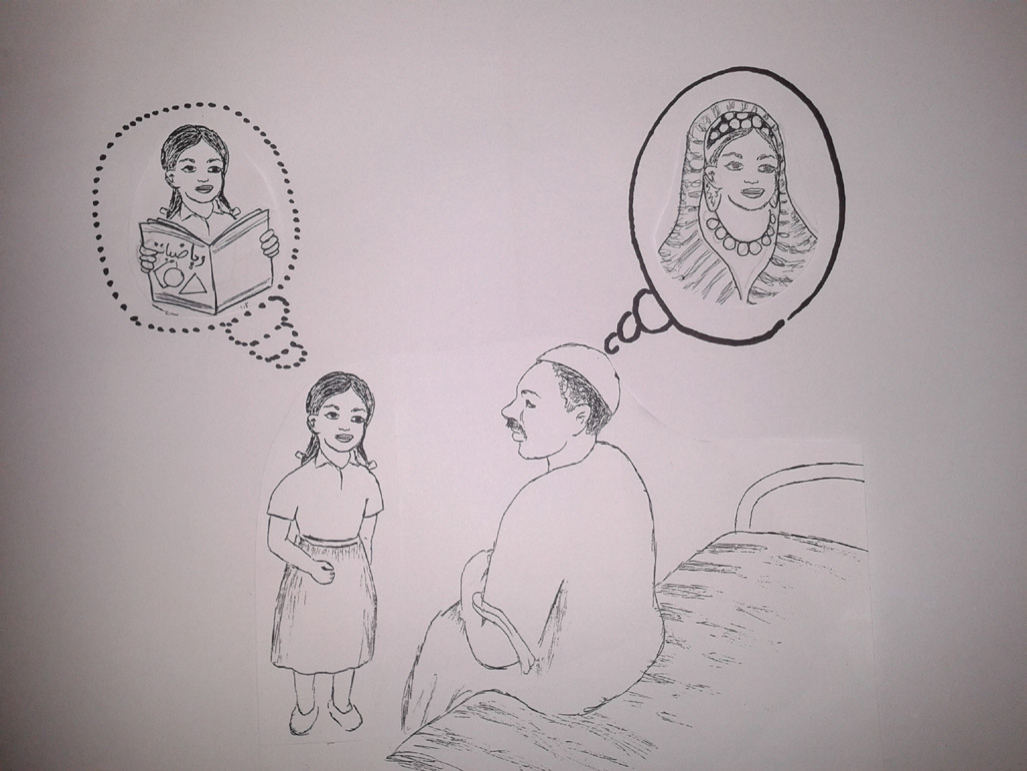
Example illustration from dissemination: limited autonomy and decision-making influence of girls and women.

Community dissemination meetings were hosted to ‘member check’ preliminary analysis^16^; this involved sharing research findings with study participants and those from the same or similar population groupings to confirm interpretations of the research team during analysis and to elicit further community reflections on the findings.^14^ Visual methods were used in this process to support the engagement of community members through the creation of a relaxed and accessible environment. Pictures were developed with a local artist to represent the major themes (figure 2) and were presented to two groups separately: (1) older women (those above the age of 25) and men, and (2) adolescent girls (married and unmarried). Community members were asked to self-group based on these categories, and therefore groupings tended to be made based on ‘social age’ within the community as opposed to ‘actual age’. Participants were asked to discuss what each picture meant, after which the research team members summarised the key messages from the study. Participants were then asked if there were any areas where they would like to see change and how they felt this could be approached. The inclusion of these meetings was approved as part of the overall study ethical approval process. Prior to commencing the meetings, all participants were informed of their purpose, given the opportunity to ask questions, and verbal consent taken for their inclusion in study analysis and presentation.

### Patient and public involvement

Participants were not involved in the design and development of this study; however, the results were disseminated to participants as described in the Community dissemination section of the manuscript.

## Results

**Figure 3:**
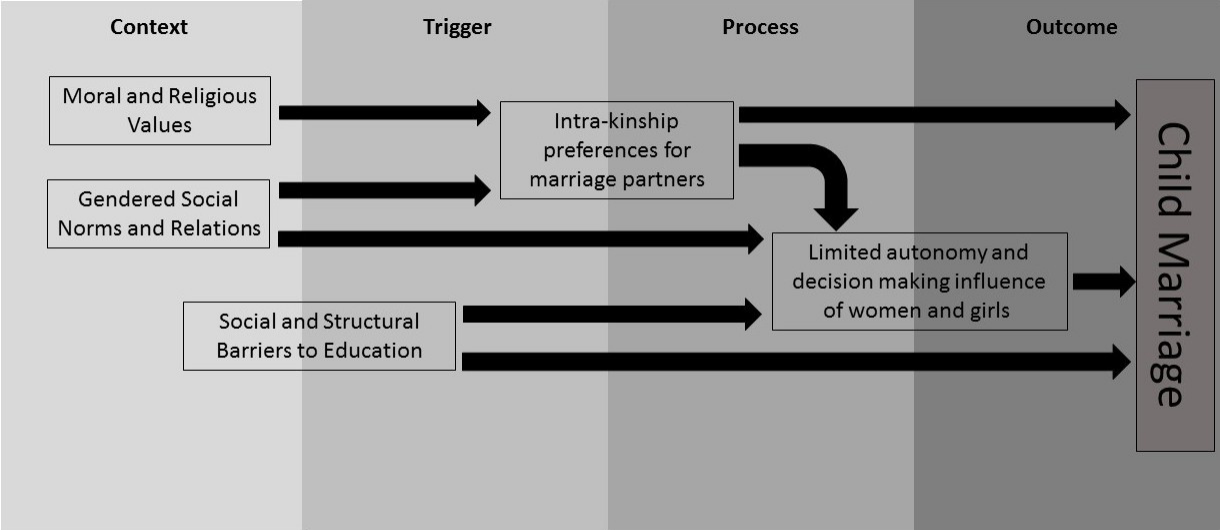
Conceptual framework for drivers of child marriage in Sudan.

Our analysis suggests that the same social norms, ideals and structural factors that shape marriage in general converge temporally to encourage child marriage in specific cases (figure 3). Theme 1 (moral and religious values) and theme 2 (gendered social norms and relations) form an underlying *context* which encourages child marriage. Elements within theme 3 (structural and social barriers to education) contribute to this underlying *context*, but predominantly act as a *trigger*, particularly when coupled with factors in theme 4 (preferences for kinship and intravillage marriage partners), that can initiate the marriage decision-making *process* via a preferred proposal. Once initiated, the marriage decision-making *process* is underpinned by influences identified in themes 4 and 5 (limited autonomy and decision-making influence of girls and women), and frequently results in the *outcome* of child marriage, which often has negative health and social consequences for women and girls (figure 3).

### Theme 1: ‘A good life’: moral and religious values shaping concepts of marriage

Moral and religious values shape ideals of marriage and criteria for marriage decision making, both directly and indirectly by underpinning gender relations. While this is the case for all marriages, themes 1 and 2 outline how these interacting values and social relations create a *context* that encourages marriages to occur at a young age.

Two key concepts were recurrent in the narratives. First, the idea of ‘sunna*’* denotes following what the prophet did, said or advised. Second is the concept of ‘khogl’ (formal Arabic) or ‘akhlag’ (colloquial Arabic), which translate as ‘moral and religious’ behaviours. In men’s narratives, sunna and akhlag position women as ‘created for marriage’; for men marriage is an obligation and demonstrates religious commitment.

The woman was created for marriage…from the man’s rib. The woman was created for the man. The Prophet told us to get married and have children. Marriage is mentioned in the Quran and Sunna. (FoB, SS2CS4)

Participants in marriage case studies stressed ‘good’ moral character and religious observance (akhlag and khulog/deen) as important characteristics to consider in deciding on the potential suitability of a groom or bride. For grooms, these are also linked closely to an individual’s social standing within the community; for brides, they are often linked to her perceived ability to fulfil the gendered role of a wife.

We didn’t refuse his proposal because we thought he was a good man (زول ود ناس)/zoul)…He is suitable for her because he is educated and he knows the Quran. (MoB, SS4CS7)

She is polite and informed and has a good moral character **ماسكة فضيلة**)/akhlag)…she respects her husband and respects us all. (FoG, SS3CS3)

Imams were described as highly influential over behavioural ideals and religious values, thus playing a significant role in shaping life aspirations, family interaction and marriage decision making.

### Theme 2: gendered social norms and relations

Gendered social norms and relations shape the aspirations and concerns of actors in marriage decisions to create a *context* which encourages earlier and consequently child marriage. Participants described strong normative gendered roles which frame the husband as a provider and protector of a subordinate wife who serves as a mother, caregiver and home maker. Gendered, moral and religious values therefore interconnect to emphasise the central role of marriage for women and girls, to the extent that remaining unmarried is stigmatised. Stigmatisation of older, unmarried girls and their families acts as a driver for families’ acceptance of a suitable proposal, regardless of their daughter’s age, for fear of missing the opportunity for marriage. Thus, the social risk for women and their families of remaining unmarried encourages earlier marriage.

Thank God, we don’t have any older girls at all, we don’t leave her without marriage. Her father and her cousins wouldn’t let her remain unmarried. Your brother’s son would marry your daughter and would never let her remain unmarried once she becomes a woman. (Ex-village committee head, SS3)

Although rarely explicit in participant accounts, the importance of female chastity to the construct of family ‘honour’ (arr’dh) underpins gender norms regarding sexuality and marriage. The stigma attached to sexual relationships outside of marriage for girls emerged through articulations of fears related to girls’ education; for example, if they continued their education, they were more likely to have to travel outside of the community, placing them at increased ‘risk’ of extramarital relationships. Thus, desire to protect ‘honour’ underpins women’s and girls’ limited autonomous spatial mobility, particularly regarding work and social interaction. One adolescent girl explained that if married, protecting a girl’s honour becomes her husband’s responsibility. Accepting a marriage proposal is thus a way of discharging the perceived family burden of protecting a girl’s chastity.

We cannot let a girl go (to school) and this is not just me, it is everyone in the rural villages. ….you have to protect your honour, don’t let your daughter get away from you, it is shameful. (FGD, MAG, SS3)

Adolescent girls described how gendered social roles and restrictions shape and limit their aspirations. Most expressed a strong desire to fulfil women’s key ascribed roles, perceiving a lack of alternatives. It is socially acceptable for out-of-school girls to contribute to household livelihoods outside the house when accompanied by family members, but only prior to marriage. While in cases of financial necessity a few wives and husbands would consider a wife working, in all narratives, married women’s potential contributions to household livelihoods were clearly positioned as secondary to fulfilling their reproductive roles.

I honestly don’t know a job to desire it in the first place. If I found a suitable job that I know how to do I’ll do it, but…I don’t know anything to do, and here girls don’t work. (MAG, SS4CS6)

Pronatalism is a key social norm and contextual factor that function to encourage earlier and thus child marriage. Grooms frequently stated a preference for younger girls, mainly because they are seen as more fertile or have potential for a longer childbearing period. In several village dissemination meetings, girls described pressure to have large numbers of children, since otherwise “people will talk and say [to the husband]…‘you can marry again…what’s the use of her, she’s not going to give you babies?’” Such preferences sometimes result in large age differences; in one case study (SS4CS9), a 25-year-old groom chose a 14-year-old girl over her older sister.

Despite recognition in some narratives that early and frequent childbearing presents threats to a girl’s health, this was rarely seen as a reason to delay marriage. Girls are expected to bear children immediately after marriage, and as often as every 2 years. When asked about family planning and contraceptives, most participants, particularly fathers and husbands, felt that the use of ‘such things’ were against moral and religious teachings (‘sunna and acklag’) and challenged gisma (fate in the sense of God’s will). A few women expressed a desire to use contraceptives, but explained that they are not readily available in rural communities.

Geographical access to maternal health services is also limited; some villages do not have access to a trained midwife, health facilities are distant, and transport prohibitively expensive and challenging (particularly during rainy season). Consequently, earlier marriage, resulting in earlier pregnancy, presents significant challenges to girls’ health and well-being.

The girl who left school when she was in grade 5 to get married…almost died during childbirth…She suffered for one whole day before giving birth. (Head teacher, SS4)

### Theme 3: structural and social barriers to education

Structural and social barriers to secondary education for girls function as both direct and indirect contextual factors encouraging child marriage, and also act as a trigger for marriage.

All participants valued some form of girls’ education, either because it was important for moral and gender socialisation, or because it could contribute to social and economic prosperity for the household, village and country. A few participants, particularly Imams, valued religious education over basic schooling. Despite this perceived value, according to participants most girls leave school after primary education (usually at 14 or 15), and marriage is then inevitable. However, it is also not uncommon for girls to leave school to get married.

We didn’t let her continue, we took her out for marriage…we took her out right in the middle of her exams. (MoB, SS4CS8)

Direct costs, such as school fees and books, and indirect costs of travelling to, or accommodation at, distant secondary schools present economic barriers to education for boys and girls. However, parents prioritise spending on boys’ education, in part because of their perceived greater livelihood opportunities on leaving school. Community dissemination highlighted strong demand for improving educational opportunities for boys and girls.

Absence of secondary schooling within villages, in a context of girls’ limited social mobility, presents social barriers for their enrolment. Some participants perceive that girls will be exposed to new customs and values if they attend school beyond the village. Additionally, many parents fear that social interaction with boys may lead to extramarital relationships, ‘harassment’, abduction or rape, all of which would threaten family ‘honour’. Thus, removing girls from school is a strategy for preserving cultural values and protecting family honour.

Here…they don’t allow the girl to go to school outside of the village…This is why girls…only finish elementary school and that is it…It is a financial reason but they also believe that the girl will bring shame and disgrace if she leaves the village. Or she will not be normal if she leaves because she will bring back different customs from outside. (FGD, MAG, SS2)

Gendered ideals and few alternative role models often mean that girls and their families lose impetus to overcome these barriers due to a lack of perceived intrinsic benefit. Protection of honour and preparation of girls for marriage are therefore normally prioritised from around the age of 15 (~8th grade), irrespective of ability to pay for education. Thus, schooling attendance does not delay or postpone marriage for most girls. Some girls felt that being able to continue their education should be a condition of marriage; however, this was rare in practice.

If they see someone graduate with a good certificate like engineering or medicine then they will be able to see the benefit of education…but we don’t have anyone here who graduated with a good certificate. (FGD, MAG, SS2)

Not continuing to secondary education not only acts as a trigger for child marriage but often limits the realisation of girls’ opportunities for strategic decision making and psychosocial well-being. Some girls who had left school expressed a sense of narrowing horizons and missed their previous social networks. In addition, girls who decided to leave school themselves often expressed a sense of regret but were unable to reverse their decision due to marriage. Where girls were granted the ability to stay in education longer, this often presented them with some room to exercise agency; however, this circumstance is rare and reliant on support within the girls’ family, which is usually based on a perception of excellent academic ability.

I dreamed about graduating from university and going to work and helping my family, I never thought about getting married or planned for my marital life, but as they say it was meant to be…it affects me deeply. (MAG, SS3CS5)

### Theme 4: preferences for marriage partners are mainly shaped by kinship and intravillage ties

The strongest *trigger* for earlier and consequently child marriages within the study communities was a strong preference for intrakinship and intravillage marriages. This was more important than direct economic gain and superseded all other considerations, including a girl’s age. This preference also strongly shaped the marriage decision-making process, particularly when combined with women’s and girls’ limited autonomy.

Maintaining and strengthening kinship and intravillage ties was the most prominent reason for making or accepting marriage proposals across all narratives. A first cousin (particularly patrilineal) is always prioritised. Second priority is given to a known relation within the wider kinship group. Stated rationales for this included knowing the suitor’s upbringing and character (akhlag); keeping inheritance within the kinship group; protecting women from divorce; and keeping adult daughters close to care for ageing parents. Suitors from the village or nearby villages are given third priority.

The man is my cousin and lives here in the village so I said it is better she marries him than someone from outside who would take her away. (MoB, SS3CS4)

This strong preference and desire to meet social expectations outweighs other potential considerations such as a girl’s age, educational status or desire to marry. This often means that regardless of age, a girl will be married because her first cousin or another relation proposes and parents perceive this as her best marriage prospect.

He is suitable for her, of course he is our relative…we know him, the son of my sister, if he was cheap we would know….I gave her to him because I couldn’t pass him up, had I told them this girl is young they would leave her and choose someone else. (MoB, SS4CS8)

Direct economic gain was noticeably unimportant to participants when discussing marriage decisions. In a few narratives, participants expressed that a daughter benefits her natal family more after marriage, as their economic and social responsibility for her shifts to her husband, but she is still expected to provide care for her parents. However, most participants explicitly stated that the economic status of a potential marriage partner is not relevant. In most cases a fixed rate bride price (*‘*mahar’) for the village is agreed at the beginning of marriage season, and although financial exchange takes place the cost of a marriage to the bride’s family outweighed the *‘*mahar*’*. Financial considerations were only mentioned as a rationale for accepting a suitor from outside the village.

### Theme 5: ‘Manufacturing consent’: limited autonomy and decision-making influence of girls and women

The limited power of women and girls acts as an enabler for child marriage. Parents often portrayed their daughters as able to exercise agency in marriage decision making, and some mothers emphasised a generational increase in decision-making power. However, girls’ narratives often emphasised constraints on this, due to patriarchal dominance in decision making. For example, one girl described her inability to refuse a proposal:

[My father] told me that [the suitor] came to us and we cannot turn him away and he told me that I have to marry him…so I married him…Personally I was thinking differently…my family were thinking of marrying me off at a young age and I had refused but my father wouldn’t let me refuse so I accepted. (MAG, SS4CS7)

In contrast, her father described her as central to the decision making:

She didn’t refuse him because there was affection and love between them from the start…It’s based on her opinion because she can find her man suitable once he proposes to her and won’t refuse him then. (FoB, SS4CS7)

Formal marriage discussions are always initiated by men, most commonly the suitor. For marriages between cousins, discussions are held between the girl’s father and his brother or the wife’s brother; thus, girls’ uncles often act as brokers.

Within case study narratives, consultation of girls in marriage decision making was presented as the norm. However, girls’ narratives and marriage case study analysis, as well as FGDs with adolescent girls, revealed that most girls are consulted at a late stage once family decision making is almost complete. In a minority of cases, suitors approached the girl first. This may reflect shifts in decision-making processes towards greater influence by girls; however, according to some key informants, a suitor may bypass a girl and ask her family directly if he fears her refusal, expecting them to pressurise her to accept.

I mean they consulted me, and since they accepted I had to accept as well, that’s why I accepted. I said to him [her grandfather] ‘did you accept or not?’ He said ‘we accept’ and I said ‘well then, I accept as well’. (MAG, SS4CS9)

Where girls were consulted and said nothing, this was taken as approval. Some girls described non-verbal strategies to express their disagreement with their marriage, such as hiding their face in public; however, none of these strategies changed the outcome. Several married girls expressed that it was easier to support their family’s decision so that they fulfilled daughterly expectations. When consulted, some mothers expressed reluctance to vocalise their own opinions, as if ‘something went wrong’ in their daughter’s marriage they feared blame for interfering with ‘gisma’ (fate).

The construction of silence as consent, late consultation in the marriage process and girls’ lack of power to effectively oppose their family’s will suggest that, even when girls are consulted, this is a process which could be characterised as the ‘manufacture of consent’ rather than offering a genuine opportunity for girls to exercise their own autonomy in relation to marriage.

## Discussion

Our findings suggest that gendered social norms and values, underpinned by religious beliefs and educational accessibility, interconnect to shape aspirations and concerns of key actors in all marriage decisions. In the *context* of these broad drivers, many child marriages are *triggered* by an intrakinship proposal and further enabled by girls’ and women’s relative lack of autonomy and influence in the marriage decision-making *process*.

The findings on key drivers broadly cohere with international knowledge on child marriage determinants.^17–24^ However, few empirical studies have conducted indepth qualitative investigation of these drivers from the perspectives of girls married as adolescents, their families and communities. Our analysis illuminates the specific operation of child marriage drivers within the state of Gezira in Sudan. In this discussion, we consider what specific drivers in our data mean in relation to the international evidence on what works to prevent child marriage. Lee-Rife *et al*’s^5^ review of this global evidence base identifies five core approaches in intervention programmes, used in isolation or in combination: (1) enhancing the accessibility and quality of formal schooling for girls; (2) fostering an enabling legal and policy framework; (3) educating and mobilising parents and community members; (4) empowering girls; and (5) offering economic support for girls and their families.

### Education: not enough in isolation

The complex relationship between marriage and education identified concurs with evidence from other contexts such as Egypt and Ethiopia in that out-of-school girls are more likely to be married due to a perceived lack of alternatives, but that removing girls from school for marriage, although less common, does occur.^13 17 25^ Based on our study, we believe that interventions designed solely to keep girls in school are unlikely to be effective at preventing child marriage in Sudan, unless other key drivers are also addressed. There is, however, a clear need to improve girls’ access to secondary education, given that economic barriers are significant in Gezira and out-of-school girls have no alternative to marriage. Material support interventions, including fees, books, uniforms and monetary incentives for parents, although not yet fully evaluated, have shown some short-term effectiveness to date at reducing child marriage in some contexts, through increasing accessibility and quality of formal schooling.^5 26^ Nevertheless, additional social norms negatively influenced girls’ access to schooling in this study. As in some other contexts (eg, Pakistan), parents are reluctant to risk family honour in expectation of limited returns on girls’ education.^15 25 27^ Addressing such factors requires livelihood development and social norm change, including increasing the visibility of educated women’s achievements as role models.

Education is widely recognised as a strategic resource for girls’ and women’s empowerment. Participants framed educational benefits in terms of gender-role socialisation, that is, education was perceived as making girls better wives. While this ‘strategic framing’ has been effective in enabling girls to attend school in the Sudanese context,^28^ it may potentially undermine efforts to prevent child marriage by reinforcing gender norms of marriage as the only socially acceptable postschool pathway for girls. However, any strategies aimed at changing rationales for girls’ education need to be developed in cognisance of how Sudanese women currently pursue their own ‘strategic interests’, that is, the rationale that works best for them in the current context.^28^

### Legal change: thinking beyond legislation

Much advocacy against child marriage focuses on developing enabling legal frameworks that increase the age of marriage to over 18 in line with the United Nations (UN) Convention on the Rights of the Child. Although Sudan has ratified the UN Convention, Article 34 of Sudan’s Personal Status Law (1991) does not reflect this, by allowing guardians (adult Muslim men of sound mind) to consent to marriage of minors (girls and boys under the age of 9 or 13, respectively).^29^ Consequently, the (elite-dominated) women’s movement in Sudan has used Islamic thought to challenge the state’s interpretation of sharia within the Personal Status Law (1991).^30^ Our findings illustrate the challenges to implementing legal change in this context, as in others.^31^ Individuals rarely knew their exact age, and marriage registrations were inconsistently recorded, supporting arguments that strengthening birth and marriage registration processes is essential to realise effective legal change.^32^

### Educating and mobilising parents and community members

Multistrategy approaches often include interventions aimed at promoting awareness of the negative consequences of child marriage among parents and community leaders.^5^ Our findings suggest that understanding potential risks does not necessarily mitigate against the practice, in line with studies elsewhere.^6^ However, since our study identifies wider community norms as key influences on marriage age, social and attitudinal change remains critical to prevent child marriage in this context.

‘Traditional values’ are often cited as the most common driver of child marriage.^6 22^ Our qualitative data elucidate the detailed dimensions of ‘traditional’ values in the Sudanese context, how the concept of family ‘honour’ and ideals of marriage are deeply embedded within religious values and teachings, in common with numerous religious and cultural settings.^6 22^ Imams were frequently cited as highly influential over family and kinship groups. Inclusion of such ‘custodians of culture’^8^ is critical in intervention development, particularly since, if overlooked, they can promote resistance to attitudinal change.^25^ The strategy of framing gender equitable social change messages within religious texts and teachings (eg, the Saleema Campaign in Sudan: https://www.unicef.org/sudan/protection6092.html) has been explored by development communication specialists^33^ and should be harnessed for social change.

Strong preferences for intrakinship and particularly consanguineous marriage were key culturally embedded triggers of child marriage. Resonating with findings from other (principally Islamic) contexts, these relate closely to the rejection of Western modernity, desire to maintain social systems and protect social and cultural capital.^7 26 34^ There is a lack of evidence on how best to address this driver. Available evidence shows that rates of consanguineous and intrakinship marriage are highest in rural populations and those with low socioeconomic status and literacy,^34^ suggesting that socioeconomic development may be necessary, although perhaps not sufficient for change.

### Empowerment of women and girls: moving beyond health consequences

Socially conservative gendered norms, underpinned by interpretations of Islam, both encourage earlier marriage and have negative implications for the health and well-being of married girls and women. The concept of ‘honour’ centres around control of women’s sexuality, while pronatalist values centre on marriage and motherhood as women’s primary role.^35^ As in other studies, use of contraception is constructed by some as unacceptable in Islam.^24^ Lack of service availability combines with women’s limited autonomy and mobility to reduce access to both contraception and maternal health services.^24^ Thus, increased reproductive health service availability is needed, but enabling women’s and girls’ social access to services and control over their own bodies requires social change. This implies the need to raise awareness of the wider negative implications of gender inequity for girls’ and women’s well-being, in addition to the health consequences of early childbearing.^36^

Married adolescent girls described a sense of isolation, which resonates with the argument that in many Islamic settings marriage is a key structure for female seclusion.^37^ There is broad evidence internationally that girls’ lack of power to decide on their own marriage and their limited autonomy within marriage have negative implications for their mental and physical health and well-being.^6^ Thus, wider initiatives to facilitate processes of ‘conscientisation’^38^ and empowerment of women and girls, enabling them to recognise and realise their rights and support their reproductive health, will be critical both to preventing child marriage and promoting wider well-being. Participatory action research has proved useful in stimulating leadership by women and simultaneously engaging men and boys to support change.^39^

### Economic support: the importance of context

Poverty is frequently cited as a critical driver of child marriage, which is seen as a strategy to reduce household economic burdens or to gain ‘bride price’.^6 8 17 21^ Cash transfers are therefore a common strategy to delay marriage.^6^ Our findings suggest that since community values and norms are much stronger than direct economic drivers, cash incentives *alone* are unlikely to be effective in this context. Nevertheless, existing evidence for links between household wealth and child marriage in Sudan^40^ suggests that broader community development strategies aimed at economic development could contribute to tackling child marriage. Alternative economic interventions often centre on developing girls’ livelihoods, since girls who participate in the labour force earlier tend to marry later.^17^ In rural Gezira, low spatial mobility and the acceptability of women’s and girls’ extra household work present challenges for this approach. However, wider community development strategies, including improving educational and livelihood opportunities for both girls and boys, may be critical to underpin social change.

### Implications for change processes in the Sudanese context

Our findings suggest the importance of embedding interventions to prevent child marriage within wider community development. As increasingly recommended by the evidence, simultaneous multisectoral strategies are required to holistically address the practice in Sudan, including expansion of access to education, health services and livelihood opportunities for both young women and men; promoting pathways of empowerment of married and unmarried young women and girls; legal change and its implementation; and participatory social and attitudinal change processes, including engaging religious leaders and men, to encourage reflection on the negative consequences of child marriage.

### Strengths and limitations of our study and implications for further research

Our study is the first to explore indepth drivers of child marriage from household and community perspectives in Sudan, and offers detailed understanding of underlying social norms and processes, to inform intervention development. Its key strengths include its holistic approach, triangulation across multiple qualitative methods and participants; use of indepth case studies to explore context, triggers and processes that result in the outcome of child marriage; and deep engagement with study communities, enabling ‘participant’ checking. However, this was a small-scale qualitative study which can only be generalised to rural Gezira State. Additional research should aim to further elucidate how drivers are shaped by intersecting social divisions such as geographical location, ethnicity and socioeconomic status to inform interventions.^35^ Due to gendered social norms, we had a high turnover of female field staff, some were unable to travel to rural contexts to collect data, and once married some were no longer able to work. While this did not substantially influence the quality of our data, it highlights the substantial challenges to conducting research on gender and women’s health in Sudan.

## Conclusion

Our study has revealed complex interactions between sociocultural, religious, gender norms and values, and educational accessibility in shaping both child marriage decisions and wider health and well-being of married adolescent girls and women. Rather than child marriages having their own unique drivers, the same social norms and structural factors that shape marriage in general converge temporally to encourage child marriage in specific cases, often triggered by an intrakinship proposal. These findings illustrate the importance of context-embedded interventions for change.^41^ We have identified several challenges facing intervention development in this context, which may limit the likely efficacy of health and social interventions that have been successful elsewhere. Ultimately, the interconnectedness of drivers demands holistic and multisectoral approaches which aim to increase girls’ and women’s age at marriage, and improve their position, health and well-being within and beyond marriage.^8^
